# Mathematical Analysis of Reaction–Diffusion Equations Modeling the Michaelis–Menten Kinetics in a Micro-Disk Biosensor

**DOI:** 10.3390/molecules26237310

**Published:** 2021-12-02

**Authors:** Naveed Ahmad Khan, Fahad Sameer Alshammari, Carlos Andrés Tavera Romero, Muhammad Sulaiman, Ghaylen Laouini

**Affiliations:** 1 Department of Mathematics, Abdul Wali Khan University, Mardan 23200, Pakistan; ahmednaveed854477@gmail.com; 2 Department of Mathematics, College of Science and Humanities in Alkharj, Prince Sattam bin Abdulaziz University, Al-Kharj 11942, Saudi Arabia; f.alshammari@psau.edu.sa; 3 COMBA R&D Laboratory, Faculty of Engineering, Universidad Santiago de Cali, Cali 76001, Colombia; carlos.tavera00@usc.edu.co; 4 College of Engineering and Technology, American University of the Middle East, Egaila 54200, Kuwait; ghaylen.laouini@aum.edu.kw

**Keywords:** micro-disk biosensor, mathematical modeling, Michaelis–Menten kinetics, enzymatic reaction, artificial neural networks, soft computing, Levenber–Marquardt training

## Abstract

In this study, we have investigated the mathematical model of an immobilized enzyme system that follows the Michaelis–Menten (MM) kinetics for a micro-disk biosensor. The film reaction model under steady state conditions is transformed into a couple differential equations which are based on dimensionless concentration of hydrogen peroxide with enzyme reaction (H) and substrate (S) within the biosensor. The model is based on a reaction–diffusion equation which contains highly non-linear terms related to MM kinetics of the enzymatic reaction. Further, to calculate the effect of variations in parameters on the dimensionless concentration of substrate and hydrogen peroxide, we have strengthened the computational ability of neural network (NN) architecture by using a backpropagated Levenberg–Marquardt training (LMT) algorithm. NNs–LMT algorithm is a supervised machine learning for which the initial data set is generated by using MATLAB built in function known as “pdex4”. Furthermore, the data set is validated by the processing of the NNs–LMT algorithm to find the approximate solutions for different scenarios and cases of mathematical model of micro-disk biosensors. Absolute errors, curve fitting, error histograms, regression and complexity analysis further validate the accuracy and robustness of the technique.

## 1. Introduction

A biosensor is a device that converts the results of biological processes into analytically accessible data. The amount of product created during the biological process affects the information analysis. Generally, the process comprises of two components named physicochemical transducer and biochemical recognition system [1,2,3]. Physical signals are generated by converting biochemical results with a specified sensitivity, which is generally considered analytic concentration, by a bioreceptor. Another component of a biosensor is transducer that produces electric signals from receptor output categorized by transducer and bioreceptor. Biosensor uses enzymes, cell structure, bioreceptors, antibodies, hormones, nucleic acid, and tissues. Auxiliary enzymes are also used in the development of biosensors. Transducers systems are categorized into thermometric, magnetic, electrochemical, and piezoelectric [4]. Biochemical reactions between an immobilized bimolecular and the target analyte form an electro-chemical biosensor. As a result, the theoretical foundation of a biosensor measures the electric current [5]. Electrochemical biosensors are categorized into potentiometric and amperometric, which are used for the mass production [6,7,8]. The functioning of an amperometric biosensor is based on calculating the Faraday current, which is determined when the current is constant at the electrode. As a result, the current is generated by the product’s oxidation or reduction [9,10,11]. Michaelis–Menten kinetic equations are commonly used to simulate the process.

In 1975, Mell [12] developed a mathematical model for amperometric two enzyme biosensors with multiple enzymes. Various numerical and analytical techniques have been used to calculate the analytical and approximate solution to the model. Che and Dong [13] reported analytical expressions for the steady-state concentration of current at the micro-disk chemical sensor. An approximate solution in integral form has been calculated by Phanthong [14] for micro-disk biosensor. Eswari derived the analytical solution in terms of enzyme kinetics and film thickness [15] for all parameters of the Michaelis–Menten constant. Manimozhi [16] study the steady-state concentration of substrate of nonlinear equations representing the action of the biosensor using homotopy perturbation method (HPM). Eswari in [15,17] 2010, derived an analytical solution for steady-state current on enzyme-modified microcylinder electrodes, micro-disk, and spherical biosensor. Loghambal [18] uses the asymptotic method (AM) to study the modeling of amperometric oxidase on enzyme membrane electrodes.

Recently, various techniques have been implemented on nonlinear ECE reactions to study the steady-state concentration of substrate and products on rotating disk electrodes [19]. M.C. Devi [20] uses hyperbolic function method (HFM) to find the analytical expression for the EC-catalytic mechanism of the first order. Variational iteration method (VIM), differential transformation method [21,21] and homotopy perturbation methods [22] has been widely used to calculate the approximate series solutions for the mathematical model of micro-disk biosensors. The implementation of these techniques has not been straightforward. For most problems, the above-discussed techniques fail to converge the solution into closed form and are time-consuming. In recent times, a stochastic numerical technique based on artificial intelligence has been developed to solve stiff nonlinear problems arising in various fields. Such stochastic computing techniques use artificial neural networks to model approximate solutions. These numerical solvers have wide applications in various fields including petroleum engineering [23], wireless communication [24], heat transfer [25,26,27], fuzzy systems [28], plasma system [29], civil engineering [30,31], wire coating dynamics [32] and Diabetic retinopathy classification [33]. The techniques mentioned earlier inspire the authors to explore and incorporate the soft computing architectures as an alternative, precise and feasible way for solving the mathematical model of micro-disk biosensors. Some highlighted features of the presented study are illustrated as follows:

- A mathematical model for micro-disk biosensor has been presented to investigate the influence of variations in different parameters on the dimensionless concentration of substrate and hydrogen peroxide.
- An artificial-neural-networks-based backpropagated Levenberg–Marquardt training (LMT) algorithm is developed to train the hidden neurons, calculate the validation of reference data-set generated by “pdex4” for different cases and scenarios of micro-disk biosensor.
- Extensive graphical analysis has been conducted based on mean square error (MSE), absolute errors, regression, curve fitting, and error histograms that show the technique’s convergence, accuracy, and computational complexity. 3D plots of dimensionless concentration for substrate (S) and hydrogen peroxide (H) are plotted against dimensionless distance *R* and reaction–diffusion parameters to study the behavior and changes in the model.

## 2. Problem Formulation

**Figure 1:**
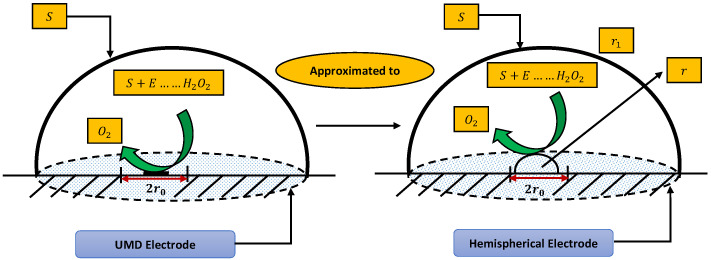
Schematic of micro-disk biosensor.

In this section, a mathematical derivation of the microdisk biosensor is presented. A generalized form of the polymer solution of drop coating is considered the special case for micro-disk electrodes. It has been observed that microdisk is insulated through droplets of enzymes or polymers, taking the form of hemisphere on isolating plane. Phanthong and Somasundrum [14] describe the mathematical formulation of a micro-disk biosensor. Figure 1 shows the schematic view of a micro-disk biosensor. It can be observed that the radius of the film is greater than the size of the disk. Measurements can be simplified by moving the micro-disk sensor to the micro-hemisphere. In [14] the micro-disk electrode is modified by using redox polymer. In such case, the enzymatic reaction favors Michaelis–Menten kinetics, and film reaction is given as

(1) $$S+E_{1}{\overset{k_{1}}{\Leftrightarrow }}_{k_{2}}\left[E_{1}S\right]\overset{k_{cat}}{\to }P+E_{2},$$

if the solution is agitated evenly such that *S* is continuously applied to the film, the mass balance at steady state for *S* will be given by

(2) $$\frac{D_{S}}{r^{2}}\frac{d}{dr}\left(r^{2}\frac{dC_{S}}{dr}\right)-\frac{k_{cat}C_{E}C_{S}}{C_{S}+K_{M}}=0,$$

where $C_{S}$, $C_{E}$ denotes the concentration profiles of substrate and enzymes, $D_{S}$ represents the diffusion coefficient of reaction, $K_{M}$ is Michaelis constant which is defined as

(3) $$K_{M}=\frac{\left(k_{-1}+k_{cat}\right)}{k_{1}},$$

steady state mass balance for *H* is given as

(4) $$\frac{D_{H}}{r^{2}}\frac{d}{dr}\left(r^{2}\frac{dC_{H}}{dr}\right)+\frac{k_{cat}C_{E}C_{S}}{C_{S}+K_{M}}=0,$$

concentration profile of steady state hydrogen peroxide is denoted by $C_{H}$. Boundary conditions at surfaces of electrode $r_{0}$ and film $r_{1}$ are defined as

$$r=r_{0}:\;\frac{dC_{S}}{dr}=0,\;C_{H}=0,$$

$$r=r_{1}:C_{S}=C_{S}^{*},\;C_{H}=0,$$

where $C_{S}^{*}$ represents the bulk concentration of *S*.

Now introducing the following set of dimensionless variables

(5) $$S=\frac{C_{S}}{C_{S}^{*}},H=\frac{C_{H}}{C_{H}^{*}},R=\frac{r_{1}}{r_{0}},\alpha =\frac{C_{S}^{*}}{K_{M}},\gamma_{E}=\frac{k_{cat}C_{E}r_{0}^{2}}{D_{S}K_{M}},\gamma_{S}=\frac{k_{cat}C_{E}r_{0}^{2}}{D_{H}K_{M}},\frac{D_{H}}{D_{S}}=\frac{\gamma_{E}}{\gamma_{S}},$$

where *S* and *H* represent the dimensionless concentration profiles of substrate and hydrogen peroxide. *R* is dimensionless distance, $\gamma_{E}$, $\gamma_{S}$ and α are reaction–diffusion and saturation parameters respectively. Thus, dimensionless form of Equations (2) and (3) along with boundary conditions can be written as

(6) $$\frac{d^{2}S}{dR^{2}}+\frac{2}{R}\frac{dS}{dR}-\frac{\gamma_{E}S}{1+\alpha S}=0,$$

(7) $$\frac{d^{2}H}{dR^{2}}+\frac{2}{R}\frac{dH}{dR}+\frac{\gamma_{S}S}{1+\alpha S}=0,$$

with

$$\frac{dS}{dR}=0,\;H=0\;when\;R=1,$$

$$S=1,\;H=0\;when\;R=r_{1}/r_{0}.$$

## 3. Reference Solutions

Various analytical and numerical techniques have been previously developed to solve the mathematical model of a micro-disk biosensor in the literature. A number of these analytical methods focus on obtaining an analytical expression for the model. These methods include variational iteration method (VIM) [34], Li-He’s variational principle methods [35,36,37], Akbari-Ganji Method (AGM Method) [38], homotopy perturbation method (HPM) [39], Modified Adomian decomposition method (MADM) [40,41], exp-function method [42], Green’s function iteration method [20] and Taylor series method [43]. An analytical expression for concentration of substrate and hydrogen peroxide obtained by HPM [15] is given as

(8) $$C_{SP}=\frac{r}{r_{0}}-0.5\frac{r^{2}}{r_{0}^{2}}+0.5\frac{r_{1}^{2}}{r_{0}^{2}}-\frac{r_{1}}{r_{0}},$$

(9) $$C_{H}P=\frac{1}{2}\left[\frac{r_{1}r}{r_{0}^{2}}-\frac{r^{2}}{r_{0}^{2}}-\frac{r_{1}}{r_{0}}+\frac{r}{r_{0}}\right],$$

where $C_{SP}=\frac{\left(c_{S}-c_{S}^{*}\right)}{c_{S}^{*}{\left(\gamma r_{0}\right)}^{2}}$ and $C_{HP}=\frac{c_{H}D_{H}}{{\left(xr_{0}\right)}^{2}D_{S}c_{s}^{*}}$. Analytical solution by MADM [40,41] are given as

(10) $$S\left(R\right)=1+\frac{\gamma_{E}}{3(1+\alpha )}\left(\frac{r_{1}}{r_{0}}\right)-\frac{\gamma_{E}}{6(1+\alpha )}{\left(\frac{r_{1}}{r_{0}}\right)}^{2}-\frac{\gamma_{E}}{3(1+\alpha )}R+\frac{\gamma_{E}}{6(1+\alpha )}R^{2},$$

(11) $$H\left(R\right)=-\frac{\gamma_{S}}{6(1+\alpha )}\left(\frac{r_{1}}{r_{0}}\right)+\frac{\gamma_{S}}{6(1+\alpha )}\left(1+r_{1}/r_{0}\right)R-\frac{\gamma_{S}}{6(1+\alpha )}R^{2}.$$

Analytical solution obtained by HAM [44] are given as

(12) $$S\left(R\right)=1+\frac{h\gamma_{E}}{2(1+\alpha )}{\left(\frac{r_{1}}{r_{0}}\right)}^{2}-\frac{h\gamma_{E}}{\left(1+\alpha \right)}\left(\frac{r_{1}}{r_{0}}\right)+\frac{h\gamma_{E}}{\left(1+\alpha \right)}R-\frac{h\gamma_{E}}{2(1+\alpha )}R^{2},$$

(13) $$H\left(R\right)=\frac{h\gamma_{S}}{2(1+\alpha )}\left(\frac{r_{1}}{r_{0}}\right)-\frac{h\gamma_{S}}{2(1+\alpha )}\left(r_{1}/r_{0}+1\right)R+\frac{h\gamma_{S}}{2(1+\alpha )}R^{2}.$$

## 4. Design Methodology

In this section, a novel machine learning technique based on supervised learning of neurons in artificial neural networks (ANNs) is utilized to study the mathematical model of an immobilized enzyme system that follows the Michaelis–Menten (MM) kinetics for micro-disk biosensor. An Artificial Neural Networks (ANNs) is a collection of interconnected, basic components known as neurons with multiple inputs and a single output, each neuron represents a mapping. The output of a neuron is a function of the sum of its inputs which is generated with the help of activation function. In this paper, multilayer perceptron (MLP) is considered, with an objective to perform the optimization of the hidden units number in the hidden layer. Additionally, the optimization of the connection weights and biases has been conducted. The typical structure of MLP with one hidden layer is given as

(14) $$S_{j}=\sum_{i=1}^{n}\omega_{ij}R+\beta_{j},$$

where, *R* denotes the input, β is a biased vector and $\omega_{ij}$ represents the connection weights. Log-sigmoid is utilized as an activation function for the Feed-forword neural network model which is given as

(15) $$f_{j}\left(x\right)=\frac{1}{1+e^{-S_{j}}},$$

**Figure 2:**
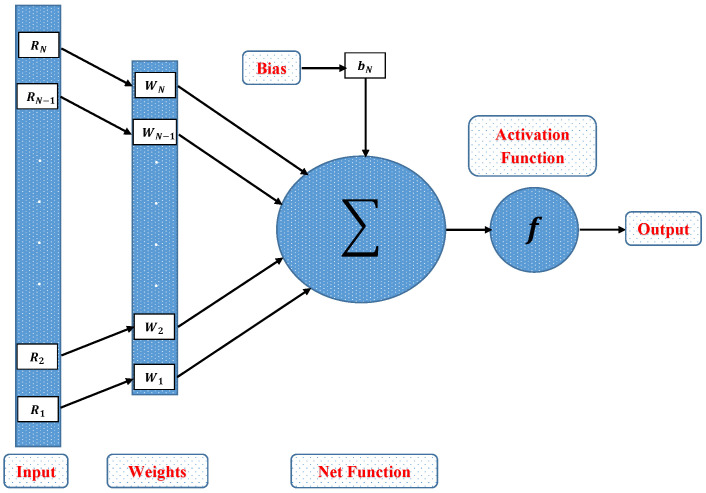
Architecture of single neuron model.

**Figure 3:**
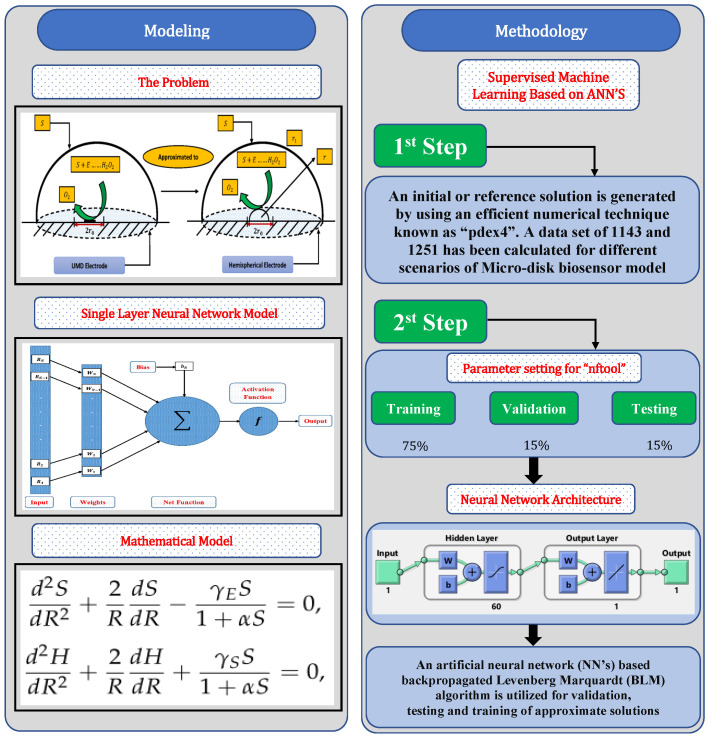
Working mechanism of design NNs–LM technique for numerical solution of mathematical model of micro-disk biosensor.

Further, the working procedure of the design soft computing technique has been discussed. Implementation of the proposed technique is based on two steps. Initially, a data set is generated for the mathematical model using an efficient numerical technique of Matlab builtin function known as “pdex4”. The date set of 1143 and 1251 points are generated from 0 to 5 and 0 to 1.5, respectively. Furthermore, an intelligent strength of neural networks with 60 hidden neurons is utilized by using the Levenberg–Marquardt technique to find approximate solutions for different scenarios and cases of the problem. The proposed NN’s-LMT algorithm in the form of a single neuron model is shown through Figure 2. The supervised learning of the Levenberg–Marquardt technique uses the data set generated in the first step by using the “nftool” package of MATLAB. The working procedure of processing data for validation and testing is shown through the flow chart given in Figure 3. Moreover, performance measures are defined in terms of mean square error of the objective function, regression study, error histograms, and absolute errors to study the accuracy and convergence of the design scheme.

## 5. Experimentation Setup and Discussion

**Figure 4:**
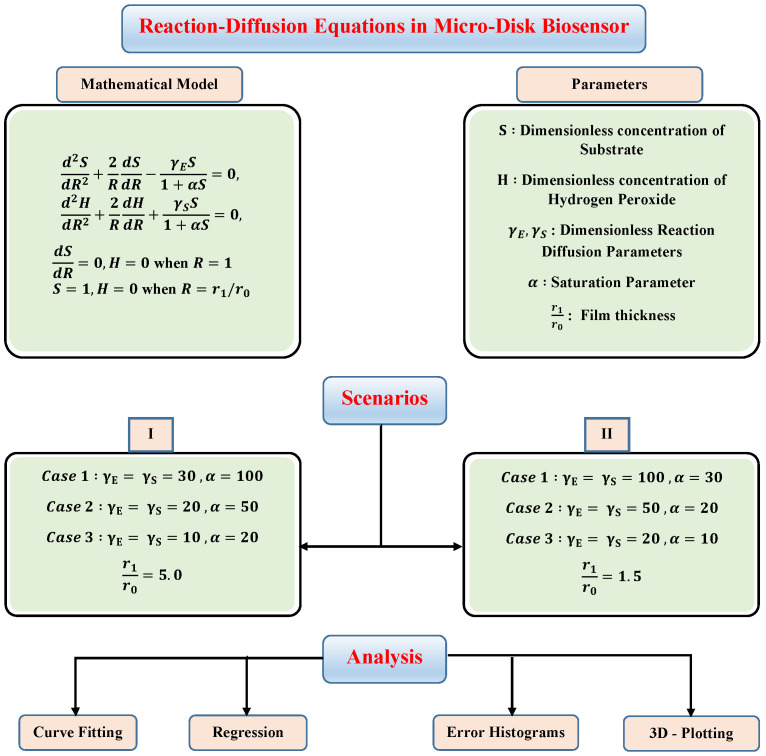
Different scenarios and cases of micro-disk biosensor.

In this section, to check the validity of the proposed technique and study the effect of variations in reaction–diffusion parameters on substrate and hydrogen peroxide concentration profiles, we have considered certain scenarios. The scenarios are based on different values of reaction–diffusion and saturation parameters with fixed values of thin-film $r_{1}/r_{0}$. The details of different scenarios and cases are dictated in the flow chart given in Figure 4.

**Figure 5:**
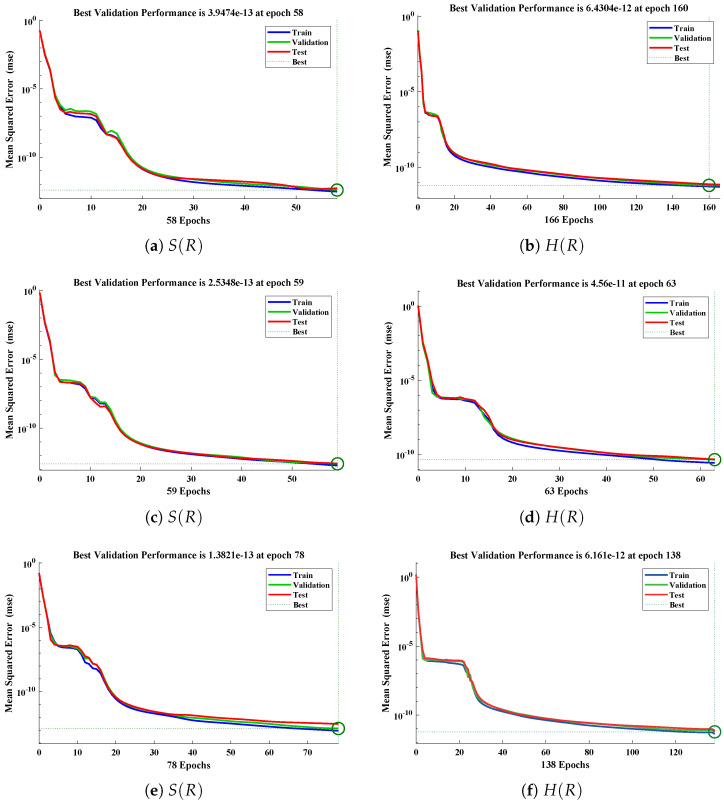
Performance of NNs–LM technique in terms of mean square error for (**a**,**c**,**e**) dimensionless concentration of substrate and (**b**,**d**,**f**) hydrogen peroxide of scenario I.

**Figure 6:**
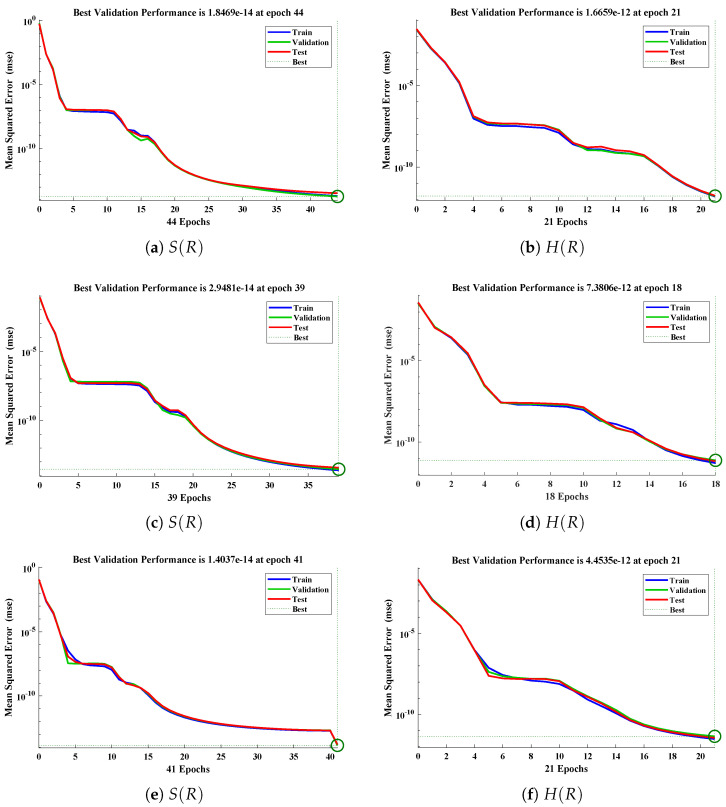
Performance of NNs–LM technique in terms of mean square error for (**a**,**c**,**e**) dimensionless concentration of substrate and (**b**,**d**,**f**) hydrogen peroxide of scenario II.

**Figure 7:**
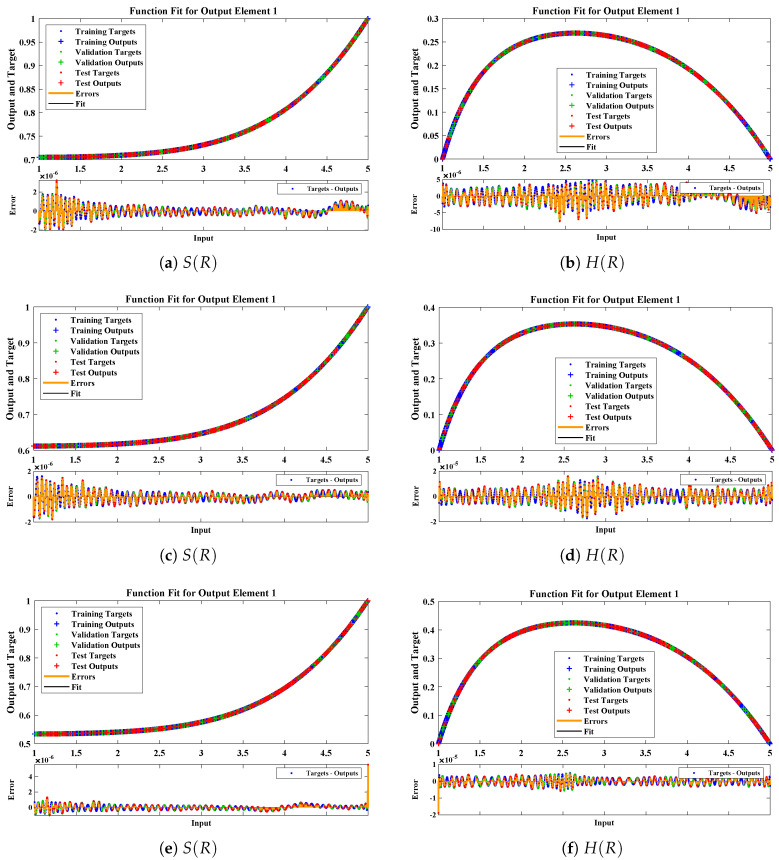
Comparison of approximate solutions obtained by NNs–LMT with numerical solution for different cases of Scenario-I.

**Figure 8:**
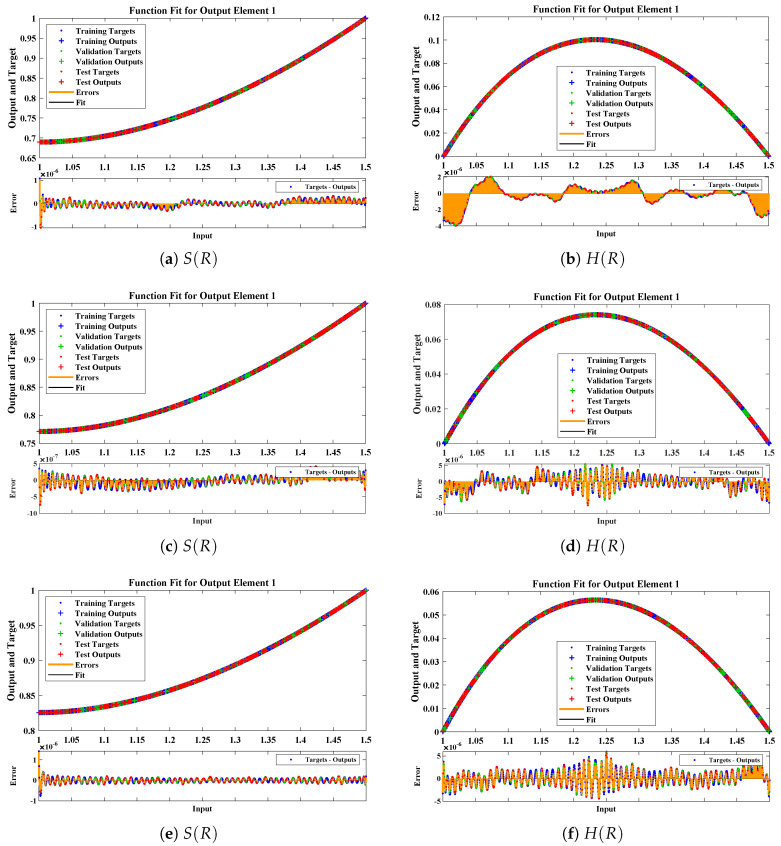
Comparison of approximate solutions obtained by NNs–LMT with numerical solution for different cases of Scenario-II.

**Figure 9:**
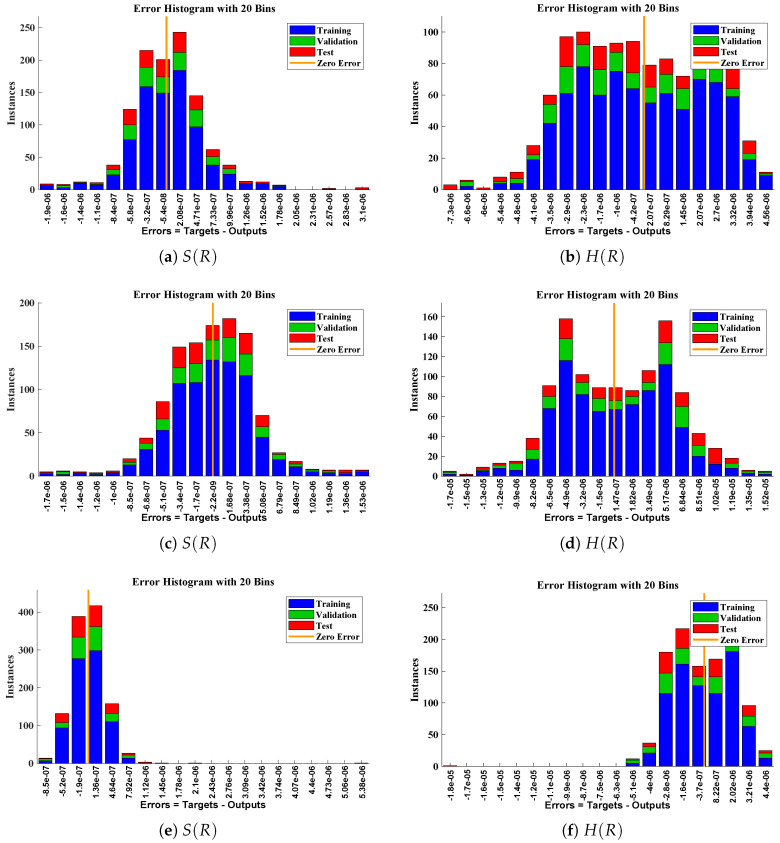
Error histogram analysis between targeted data and approximate solutions for scenario I.

**Figure 10:**
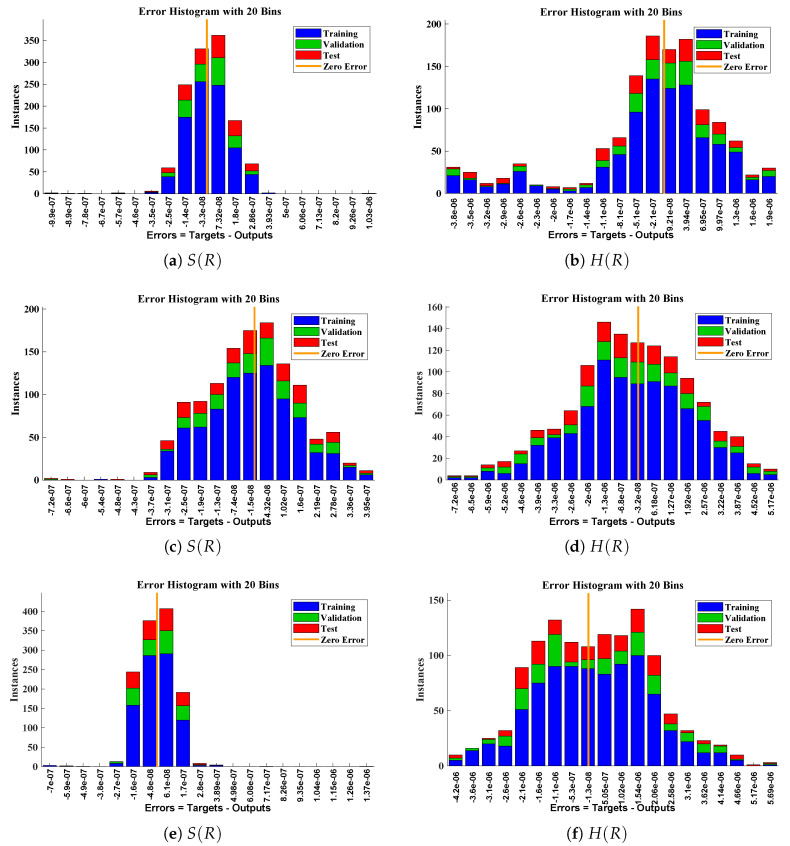
Error histogram analysis between targeted data and approximate solutions for scenario II.

**Figure 11:**
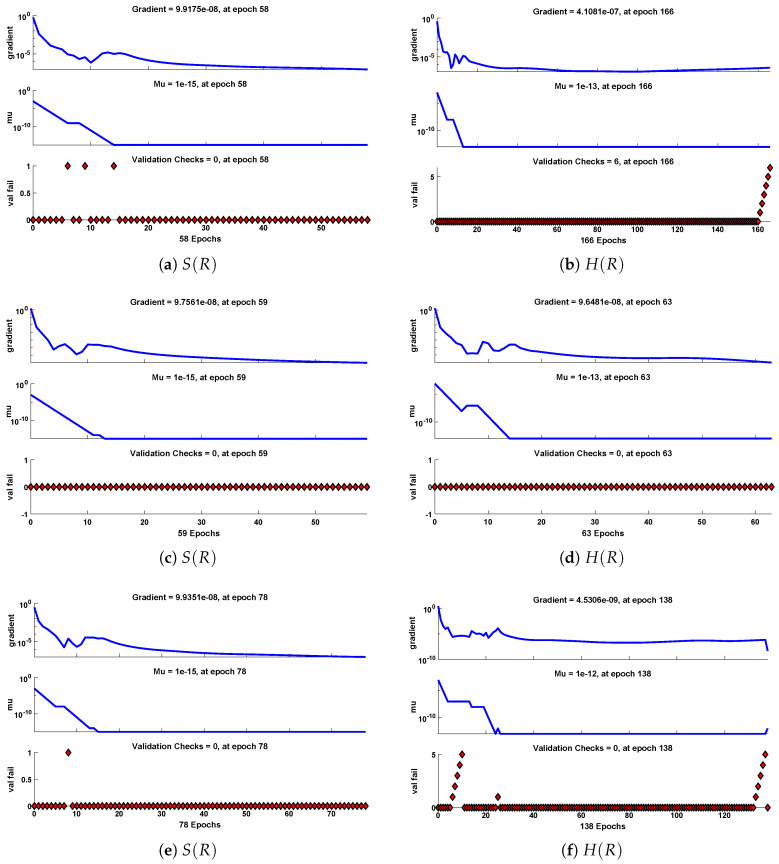
Performance of NNs–LM based on gradient, mu and validations failure during the process of optimization for different cases of Scenario I.

**Figure 12:**
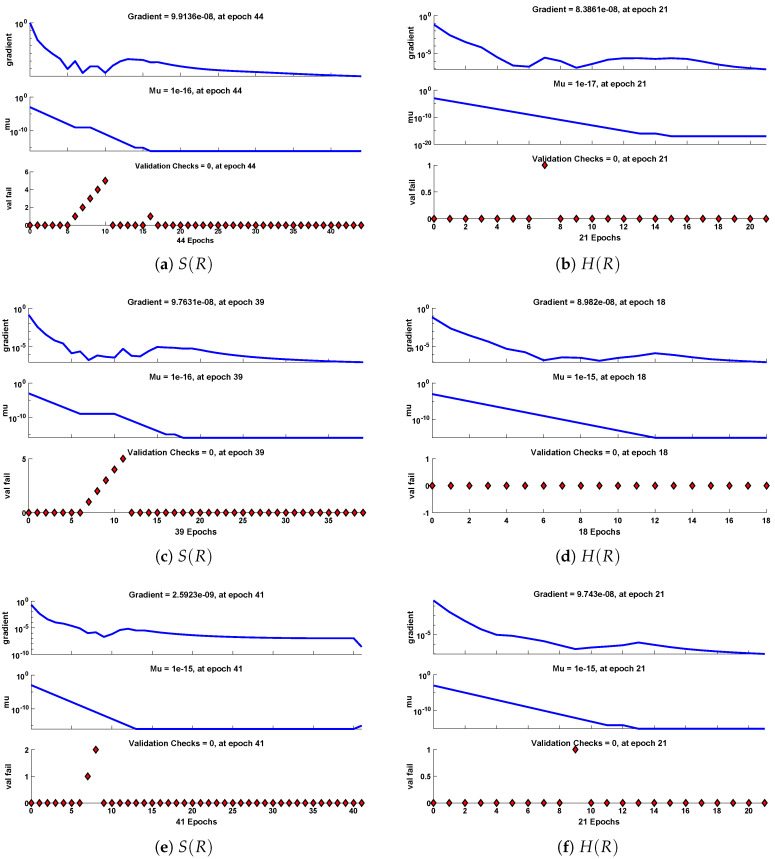
Performance of NNs–LM based on gradient, mu and validations failure during the process of optimization for different cases of Scenario II.

**Figure 13:**
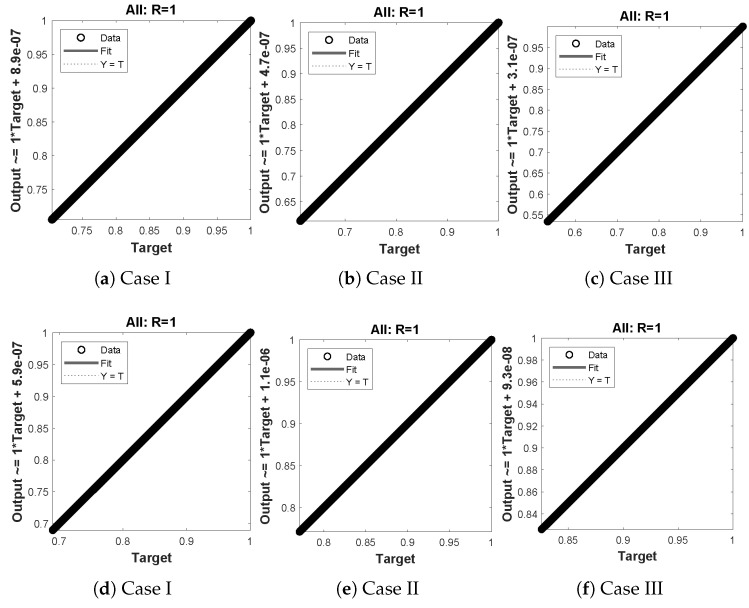
(**a**–**f**) shows the regressionanalysis for dimensionless concentration of substrate of micro-disk biosensor model.

**Figure 14:**
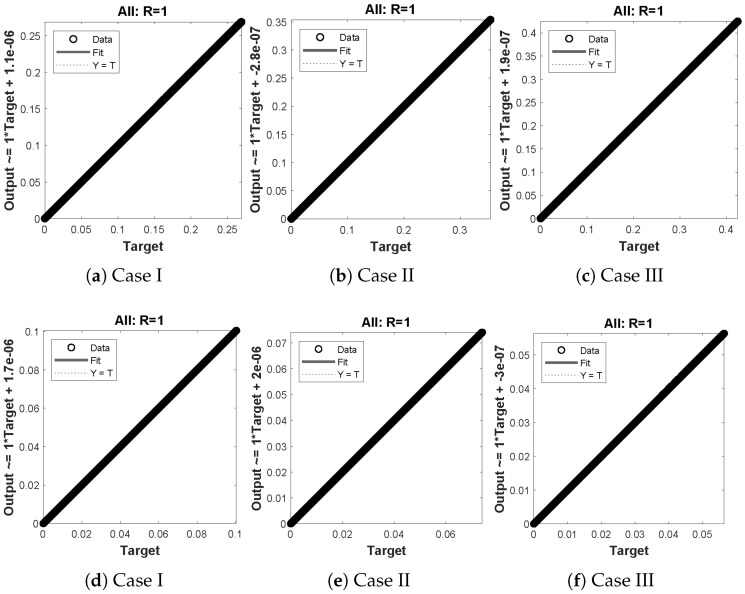
(**a**–**f**) shows the regression analysis for dimensionless concentration of hydrogen peroxide of micro-disk biosensor model.

The data set values generated by the numerical technique are used to test, train and validate the data with a probability of 75%, 15%, and 15%, respectively. Figure 5 and Figure 6 represent the convergence of mean square error (MSE) function for scenarios I and II, respectively. The performance values for each case of different scenario lies around $3.9474\times 10^{-13}$, $6.4304\times 10^{-12}$, $2.5348\times 10^{-13}$, $4.56\times 10^{-11}$, $1.3821\times 10^{-13}$, $6.161\times 10^{-12}$, $1.8469\times 10^{-14}$, $1.6659\times 10^{-12}$, $2.9481\times 10^{-14}$, $7.3806\times 10^{-12}$, $1.4037\times 10^{-14}$ and $4.4535\times 10^{-14}$. Figure 7 and Figure 8 are plotted to investigate the influence of variations in saturation and reaction–diffusion parameters with fixed values of film thickness. These parameters describe the importance of reaction and diffusion in the enzyme layer. It is evident from the figures that the concentration profile of substrate significantly increases when the values of reaction–diffusion $\left(\gamma_{E}\right)$ and saturation parameter α are increased. Additionally, a simultaneous increase is observed in the concentration of hydrogen peroxide and substrate as $\gamma_{S}$ and α decreases. The approximate solutions for concentration of substrate obtained by design algorithm are compared with the homotopy analysis method (HAM) [44], modified Adomain decomposition method [40], hyperbolic function method [45,46] and numerical solutions by Pdex4 as shown in Table 1. The fitting of approximate solution and targeted date overlap each other with minimum absolute errors as shown in the Figure 9 and Figure 10. The values of absolute errors in solutions for S(R) lies around $10^{-6}$ to $10^{-8}$, $10^{-6}$ to $10^{-9}$, $10^{-6}$ to $10^{-8}$, $10^{-7}$ to $10^{-8}$, $10^{-6}$ to $10^{-8}$ and $10^{-7}$ to $10^{-8}$ respectively. Additionally, AE for the solution concentration of hydrogen peroxide lies around $10^{-6}$ to $10^{-7}$, $10^{-5}$ to $10^{-7}$, $10^{-6}$ to $10^{-7}$, $10^{-6}$ to $10^{-8}$, $10^{-6}$ to $10^{-8}$ and $10^{-7}$ to $10^{-8}$. The statistical performance in term of gradient, mu, validation failures and regression analysis are plotted in Figure 11, Figure 12, Figure 13 and Figure 14 and dictated in Table 2 and Table 3 respectively. The value gradient for all the cases of micro-disk biosensor are $9.92\times 10^{-13}$, $4.11\times 10^{-7}$, $9.76\times 10^{-8}$, $9.65\times 10^{-8}$, $9.94\times 10^{-8}$, $4.53\times 10^{-9}$, $9.91\times 10^{-8}$, $8.39\times 10^{-8}$, $9.76\times 10^{-8}$, $8.98\times 10^{-8}$, $2.59\times 10^{-9}$ and $9.74\times 10^{-8}$.

**Figure 15:**
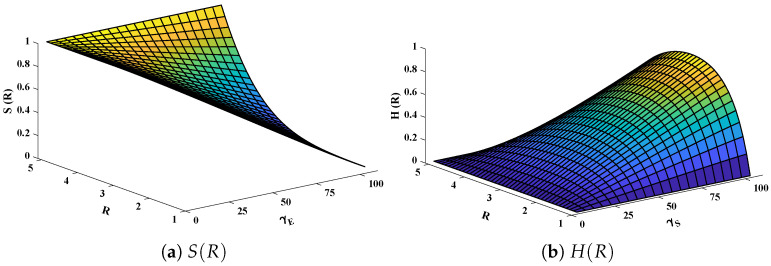
Plotof three dimensional (**a**) concentration profiles of substrate and (**b**) hydrogen peroxide against dimensionless distance *R* and reaction–diffusion parameters. Saturation parameter α is equal to 50.

**Figure 16:**
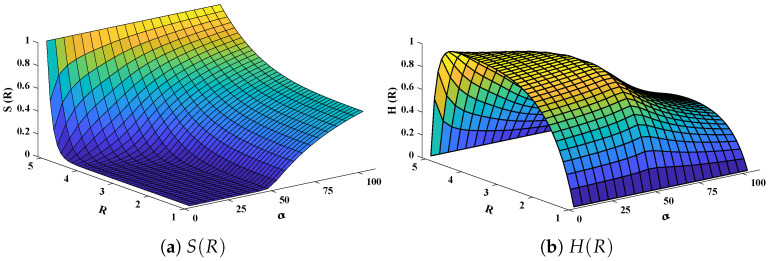
Plot of three dimensional (**a**) concentration profiles of substrate and (**b**) hydrogen peroxide against dimensionless distance *R* and Saturation parameter α. Reaction–diffusion parameters are equal to 50.

**Figure 17:**
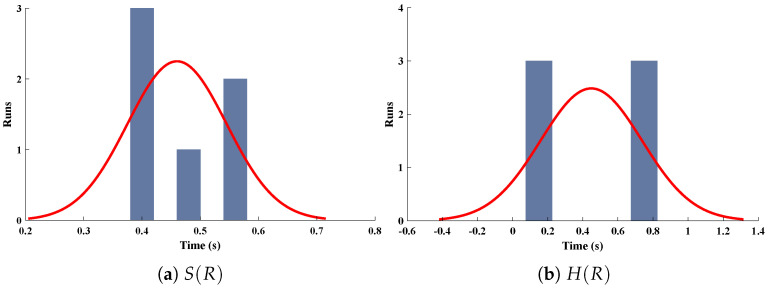
Normal probability curves for the computational complexity of different scenarios for (**a**) concentration profiles of substrate and (**b**) hydrogen peroxide of micro-disk biosensor.

Three-dimensional plots of substrate and hydrogen peroxide are plotted against dimensionless distance *R*, reaction–diffusion parameters, and saturation parameters as shown in Figure 15 and Figure 16 respectively. From the figures, the influence of variations in parameters can be observed. From Figure 15a,b, the increase in reaction–diffusion parameters $\left(\gamma_{E},\gamma_{S}\right)$ with fixed α=50 causes a decrease in the concentration profile of substrate. In contrast, a significant increase is observed in the concentration profile of hydrogen peroxide. Figure 16 represents the effect of variations in α. It is evident that slight increase in S(R) is observed till α=40 but increases sharply for α>40. Simultaneously, a decrease in the concentration of hydrogen peroxide is observed for R≠1. Normal probability curves are plotted to study the computational complexity. The time taken by the system for obtaining solutions for each scenario lies around 0.45 and 0.50 s, as shown in Figure 17.

To check the performance and validity of the proposed ANN–GNDO–SQP algorithm, we defined different statistical operators along with their global form. The performance operators are fitness functions, Theil’s inequality coefficient (TIC), mean absolute deviations (MAD), Nash Sutcliffe efficiency (NSE), and error in Nash Sutcliffe efficiency (ENSE). A mathematical formulation of these indices is defined as

(16) $$\mathrm{MAD}=\frac{1}{N}\sum_{m=1}^{N}\left|S_{m}\left(R\right)-{\hat{S}}_{m}\left(R\right)\right|,$$

(17) $$\mathrm{TIC}=\frac{\sqrt{\frac{1}{N}\sum_{n=1}^{N}{\left(S_{m}\left(R\right)-{\hat{S}}_{m}\left(R\right)\right)}^{2}}}{\left(\sqrt{\frac{1}{N}\sum_{m=1}^{N}{\left(S_{m}\left(R\right)\right)}^{2}}+\sqrt{\frac{1}{N}\sum_{m=1}^{N}{\left({\hat{S}}_{m}\left(R\right)\right)}^{2}}\right)},$$

(18) $$\;\mathrm{NSE}\;=\left\{1-\frac{\sum_{m=1}^{N}{\left(S_{m}\left(H\right)-{\hat{S}}_{m}\left(H\right)\right)}^{2}}{\sum_{m=1}^{N}{\left((S_{m}\left(H\right)-{\underline{\hat{S}}}_{m}\left(H\right)\right)}^{2}},\;{\underline{\hat{S}}}_{m}\left(H\right)=\frac{1}{N}\sum_{m=1}^{N}\hat{S}\left(H\right),\right.$$

(19) $$\;\mathrm{ENSE}\;=\left(1-\mathrm{NSE}\right).$$

where, $S_{m}$ is analytical solution and ${\hat{S}}_{m}$ represents the approximate solution by proposed algorithm. *N* denotes the grid points.

The stability, efficiency and accuracy of the proposed algorithm is established by executing the design algorithms for twenty multiple runs. Global values of performance function in terms of mean square error for each scenario lies around $3.8110^{-10}$ to $3.7510^{-12}$ and $1.1410^{-11}$ to $2.7510^{-13}$ with standard deviations $5.8910^{-9}$ to $1.0710^{-12}$ and $1.9810^{-11}$ to $3.1610^{-13}$ respectively. In addition, from Table 4 and Table 5 the values of MAD, TIC and ENSE are also approaching zero, which shows the perfect modeling of the approximate solutions by the design algorithm.

## 6. Conclusions

This paper investigated the mathematical model of an immobilized enzyme system that follows the Michaelis–Menten (MM) kinetics for micro-disk biosensors. The model was based on reaction–diffusion phenomena, which was given by a system of nonlinear differential equations. Furthermore, a soft computing technique based on supervised learning of Levenberg–Marquardt backpropagation neural networks is used to calculate substrate and hydrogen peroxide concentration under the influence of variations in several parameters, including reaction diffusion and film thickness, and saturation parameter. The results illustrated in the figures conclude that increase in reaction–diffusion and saturation parameters causes a decrease in substrate concentration while an increase in the concentration profile of hydrogen peroxide. It is also concluded that an increase in saturation parameter directly relates to substrate concentration, while inversely relating to the concentration of hydrogen peroxide. An extensive graphical analysis based on MSE, error histogram, absolute errors, regressions, and computational complexity are conducted, showing the robustness, accuracy, and efficiency of the designed scheme. 

## Figures and Tables

**Table 1:** Comparison of approximate solutions obtained by NNs–LM algorithm with homotopy analysis method, modified Adomain decomposition method, hyperbolic function method and numerical solver Pdex4.

	α=100, $\gamma_{E}=30$					α=50, $\gamma_{E}=20$					α=10, $\gamma_{E}=10$				
*R*	HAM	HFM	MADM	Numerical	NNs–LMT	HAM	HFM	MADM	Numerical	NNs–LMT	HAM	HFM	MADM	Numerical	NNs–LMT
1.0	0.7038	0.7013	0.7025	0.7003	0.7003	0.6102	0.6000	0.6110	0.6010	0.6010	0.5316	0.5310	0.5320	0.5300	0.5300
1.5	0.7109	0.7074	0.7099	0.7075	0.7075	0.6014	0.6070	0.6121	0.6072	0.6072	0.5432	0.5397	0.5390	0.5398	0.5398
2.0	0.7112	0.7103	0.7108	0.7105	0.7105	0.6123	0.6081	0.6129	0.6080	0.6080	0.5473	0.5418	0.5454	0.5420	0.5420
2.5	0.7196	0.7159	0.7177	0.7158	0.7158	0.6158	0.6132	0.6145	0.6125	0.6125	0.5467	0.5474	0.5498	0.5476	0.5476
3.0	0.7332	0.7326	0.7329	0.7327	0.7327	0.6297	0.6282	0.6298	0.6280	0.6280	0.5678	0.5650	0.5698	0.5651	0.5651
3.5	0.7565	0.7590	0.7662	0.7595	0.7595	0.6671	0.6621	0.6701	0.6621	0.6621	0.6089	0.6035	0.6094	0.6037	0.6037
4.0	0.8132	0.8131	0.8132	0.8131	0.8131	0.7219	0.7289	0.7324	0.7289	0.7289	0.6780	0.6794	0.6768	0.6795	0.6795
4.5	0.8896	0.8894	0.8895	0.8894	0.8894	0.8190	0.8260	0.8270	0.8260	0.8260	0.7936	0.7905	0.7897	0.7905	0.7905
5.0	1	1	1	1	1	0.9967	0.9965	0.9963	1	1	0.9999	0.9993	0.9998	0.9992	0.9992

**Table 2:** Statistical analysis of performance measures including MSE, Gradient, mu, number of iterations and time taken by the system for obtaining the results of Scenario I.

	Case I		Case II		Case III	
	S(R)	H(R)	S(R)	H(R)	S(R)	H(R)
Hidden Neurons	60	60	60	60	60	60
Training	$3.02\times 10^{-13}$	$5.44\times 10^{-12}$	$1.91\times 10^{-13}$	$2.76\times 10^{-11}$	$9.21\times 10^{-14}$	$8.53\times 10^{-12}$
Validation	$3.95\times 10^{-13}$	$6.43\times 10^{-12}$	$2.53\times 10^{-13}$	$4.56\times 10^{-11}$	$1.38\times 10^{-13}$	$6.16\times 10^{-12}$
Testing	$5.29\times 10^{-13}$	$7.67\times 10^{-12}$	$2.68\times 10^{-13}$	$4.76\times 10^{-11}$	$3.11\times 10^{-13}$	$5.12\times 10^{-13}$
Gradient	$9.92\times 10^{-13}$	$4.11\times 10^{-7}$	$9.76\times 10^{-8}$	$9.65\times 10^{-8}$	$9.94\times 10^{-8}$	$4.53\times 10^{-9}$
Mu	$1.00\times 10^{-15}$	$1.00\times 10^{-15}$	$1.00\times 10^{-15}$	$1.00\times 10^{-13}$	$1.00\times 10^{-13}$	$1.00\times 10^{-12}$
Epochs	58	166	59	63	78	138
Regression	1	1	1	1	1	1
Time (s)	<1 s	<1 s	<1 s	<1 s	<1 s	<1 s

**Table 3:** Statistical analysis of performance measures including MSE, Gradient, mu, number of iterations and time taken by the system for obtaining the results of Scenario II.

	Case I		Case II		Case III	
	S(R)	H(R)	S(R)	H(R)	S(R)	H(R)
Hidden Neurons	60	60	60	60	60	60
Training	$2.03\times 10^{-14}$	$1.55\times 10^{-12}$	$2.47\times 10^{-14}$	$4.98\times 10^{-12}$	$1.61\times 10^{-14}$	$3.02\times 10^{-12}$
Validation	$1.85\times 10^{-14}$	$1.67\times 10^{-12}$	$2.95\times 10^{-14}$	$7.38\times 10^{-12}$	$1.40\times 10^{-14}$	$4.45\times 10^{-12}$
Testing	$3.31\times 10^{-14}$	$1.75\times 10^{-12}$	$3.84\times 10^{-14}$	$6.51\times 10^{-12}$	$1.67\times 10^{-13}$	$3.58\times 10^{-12}$
Gradient	$9.91\times 10^{-8}$	$8.39\times 10^{-8}$	$9.76\times 10^{-8}$	$8.98\times 10^{-8}$	$2.59\times 10^{-9}$	$9.74\times 10^{-8}$
Mu	$1.00\times 10^{-16}$	$1.00\times 10^{-17}$	$1.00\times 10^{-16}$	$1.00\times 10^{-15}$	$1.00\times 10^{-15}$	$1.00\times 10^{-15}$
Epochs	44	21	39	18	41	21
Time (s)	<1 s	<1 s	<1 s	<1 s	<1 s	<1 s

**Table 4:** Stability analysis on the values of performance function and performance indicators in terms of minimum, mean and standard deviations for different cases of scenario-I.

			MSE			MAD			TIC			ENSE	
		Min.	Mean	Std.	Min.	Mean	Std.	Min.	Mean	Std.	Min.	Mean	Std.
Case I	S(R)	$3.95\times 10^{-13}$	$3.75\times 10^{-12}$	$4.86\times 10^{-12}$	$6.54\times 10^{-6}$	$1.23\times 10^{-5}$	$4.42\times 10^{-5}$	$1.96\times 10^{-7}$	$3.78\times 10^{-7}$	$1.76\times 10^{-7}$	$7.23\times 10^{-14}$	$2.81\times 10^{-13}$	$1.72\times 10^{-13}$
	H(R)	$6.43\times 10^{-12}$	$4.27\times 10^{-11}$	$3.71\times 10^{-11}$	$2.55\times 10^{-6}$	$2.22\times 10^{-5}$	$6.74\times 10^{-5}$	$2.35\times 10^{-8}$	$5.35\times 10^{-6}$	$2.88\times 10^{-7}$	$6.35\times 10^{-13}$	$9.17\times 10^{-12}$	$5.32\times 10^{-13}$
Case II	S(R)	$2.53\times 10^{-13}$	$1.13\times 10^{-12}$	$4.60\times 10^{-12}$	$1.06\times 10^{-6}$	$6.75\times 10^{-5}$	$3.06\times 10^{-6}$	$4.64\times 10^{-8}$	$4.51\times 10^{-6}$	$4.95\times 10^{-6}$	$5.05\times 10^{-14}$	$4.97\times 10^{-13}$	$2.41\times 10^{-13}$
	H(R)	$4.56\times 10^{-11}$	$3.81\times 10^{-10}$	$5.89\times 10^{-9}$	$6.92\times 10^{-6}$	$4.40\times 10^{-5}$	$3.18\times 10^{-5}$	$6.23\times 10^{-7}$	$1.36\times 10^{-8}$	$6.25\times 10^{-7}$	$1.18\times 10^{-13}$	$1.67\times 10^{-12}$	$2.10\times 10^{-13}$
Case III	S(R)	$1.38\times 10^{-13}$	$6.51\times 10^{-12}$	$1.07\times 10^{-12}$	$2.08\times 10^{-7}$	$6.17\times 10^{-6}$	$2.20\times 10^{-6}$	$4.25\times 10^{-7}$	$4.51\times 10^{-6}$	$3.35\times 10^{-6}$	$2.09\times 10^{-15}$	$7.95\times 10^{-14}$	$1.59\times 10^{-13}$
	H(R)	$6.16\times 10^{-12}$	$8.96\times 10^{-11}$	$1.23\times 10^{-11}$	$7.56\times 10^{-6}$	$8.64\times 10^{-5}$	$3.38\times 10^{-6}$	$1.49\times 10^{-7}$	$2.19\times 10^{-6}$	$2.49\times 10^{-7}$	$2.75\times 10^{-13}$	$2.95\times 10^{-12}$	$9.41\times 10^{-12}$

**Table 5:** Stability analysis on the values of performance function and performance indicators in terms of minimum, mean and standard deviations for different cases of scenario-II.

			MSE			MAD			TIC			ENSE	
		Min.	Mean	Std.	Min.	Mean	Std.	Min.	Mean	Std.	Min.	Mean	Std.
Case I	S(R)	$1.84\times 10^{-14}$	$3.40\times 10^{-12}$	$5.87\times 10^{-11}$	$1.37\times 10^{-6}$	$9.08\times 10^{-6}$	$2.12\times 10^{-6}$	$1.97\times 10^{-7}$	$1.49\times 10^{-6}$	$7.33\times 10^{-7}$	$3.10\times 10^{-14}$	$2.76\times 10^{-13}$	$2.13\times 10^{-14}$
	H(R)	$1.66\times 10^{-12}$	$1.14\times 10^{-11}$	$1.98\times 10^{-11}$	$2.58\times 10^{-6}$	$2.83\times 10^{-5}$	$2.68\times 10^{-6}$	$2.15\times 10^{-7}$	$1.28\times 10^{-6}$	$2.06\times 10^{-7}$	$9.32\times 10^{-14}$	$1.97\times 10^{-13}$	$2.75\times 10^{-13}$
Case II	S(R)	$2.95\times 10^{-14}$	$2.75\times 10^{-13}$	$3.16\times 10^{-13}$	$3.13\times 10^{-6}$	$2.66\times 10^{-5}$	$1.82\times 10^{-6}$	$7.44\times 10^{-8}$	$4.11\times 10^{-6}$	$1.46\times 10^{-6}$	$2.24\times 10^{-15}$	$2.63\times 10^{-14}$	$2.56\times 10^{-14}$
	H(R)	$7.38\times 10^{-12}$	$9.12\times 10^{-11}$	$1.80\times 10^{-11}$	$2.34\times 10^{-6}$	$2.44\times 10^{-5}$	$2.08\times 10^{-6}$	$1.30\times 10^{-7}$	$7.59\times 10^{-7}$	$8.68\times 10^{-7}$	$1.87\times 10^{-13}$	$2.55\times 10^{-12}$	$2.79\times 10^{-13}$
Case III	S(R)	$1.40\times 10^{-14}$	$7.43\times 10^{-13}$	$4.49\times 10^{-13}$	$1.56\times 10^{-6}$	$2.37\times 10^{-5}$	$2.72\times 10^{-6}$	$2.56\times 10^{-7}$	$1.90\times 10^{-6}$	$8.85\times 10^{-7}$	$2.85\times 10^{-14}$	$1.37\times 10^{-13}$	$2.14\times 10^{-13}$
	H(R)	$4.45\times 10^{-12}$	$2.69\times 10^{-11}$	$1.03\times 10^{-11}$	$2.23\times 10^{-6}$	$3.10\times 10^{-5}$	$3.44\times 10^{-6}$	$3.19\times 10^{-7}$	$2.38\times 10^{-6}$	$1.15\times 10^{-6}$	$2.73\times 10^{-13}$	$1.61\times 10^{-12}$	$2.61\times 10^{-12}$

## Data Availability

The data that support the findings of this study are available from the corresponding author upon reasonable request.
